# Prevalence of *pfmdr1* alleles associated with artemether-lumefantrine tolerance/resistance in Maputo before and after the implementation of artemisinin-based combination therapy

**DOI:** 10.1186/1475-2875-13-300

**Published:** 2014-08-06

**Authors:** Elsa Lobo, Bruno de Sousa, Soraia Rosa, Paula Figueiredo, Lis Lobo, Sara Pateira, Natercia Fernandes, Fatima Nogueira

**Affiliations:** Faculdade de Medicina, Departamento de CiênciasFisiológicas, Universidade Eduardo Mondlane, Av Salvador Allende 702, Maputo, Moçambique; Faculdade de Psicologia e de Ciências da Educação, Universidade de Coimbra, Rua do Colégio Novo, Apartado 6153, 3001-802 Coimbra, Portugal; Unidade de ParasitologiaMédica, Instituto de Higiene e Medicina Tropical (IHMT), Universidade Nova de Lisboa, Rua da Junqueira 100, 1349-008 Lisbon, Portugal; Centro de Malária e outrasDoençasTropicais (CMDT), IHMT/UNL, Rua da Junqueira 100, 1349-008 Lisbon, Portugal; Faculdade de Medicina, Departamento de Pediatria, Universidade Eduardo Mondlane, Av Salvador Allende 702, Maputo, Moçambique

**Keywords:** Malaria, Mozambique, *pfmdr1*, ACT

## Abstract

**Background:**

Mozambique implemented artemisinin-based combinations therapy (ACT) using artemether-lumefantrine (AL) as the first-line treatment for uncomplicated malaria in 2009. AL remains highly efficacious, but widespread use may soon facilitate emergence of artemisinin tolerance/resistance. The prevalence of *pfmdr1* different alleles in Maputo and Mozambique is not known, either after or before the introduction of ACT. *Pfmdr1* molecular markers related to *Plasmodium falciparum* susceptibility were analysed before and after transition to ACT.

**Methods:**

A first group of samples was collected between June 2003 and June 2005 and a second group in the period between March 2010 and March 2012. Three alleles were analysed by PCR-RFLP: N86Y, Y184F and D1246Y, in the *pfmdr1* gene.

**Results:**

Alleles N86, 184F and D1246 increased from 19.5, 19.6 and 74.4% in 2003–2005 to 73.2, 22.7 and 96.7% in 2010–2012, respectively. After implementation of ACT (2010–2012), *pfmdr1* haplotypes, either two- and three-codon basis, were generally less diverse than before the implementation of ACT (2003–2005). The prevalence of haplotypes N86-184Y, N86-D1246 and 184Y-D1246 increased from 12,2, 27.3 and 71.7% in 2003–2005 to 59.4, 84.3 and 78.6% in 2010–2012. The three-codon basis haplotypes NFD and NYD also increased significantly during the same period.

**Conclusion:**

The alleles N86 and 184 F and the triple haplotype N86-184 F-D1246 showed a significantly increased prevalence after introduction of ACT.

## Background

The *Plasmodium falciparum* multi-drug resistance gene 1 (*pfmdr1*) and particularly, single nucleotide polymorphisms (SNPs) resulting in an amino acid change in codons 86 (N86Y), 184 (Y184F), and 1246 (D1246Y) have been associated with changes in parasite susceptibility to various drugs, including artemisinin-based combination therapy (ACT) [1–4]. Initially, SNPs in *pfmdr1* were associated with chloroquine (CQ) and amodiaquine (AQ) resistance [5]. For instance, the *pfmdr1* 86Y mutation has been associated with high CQ resistance [6, 7], and the combination of *pfmdr1* 86Y, Y184, and 1246Y is likely selected by AQ monotherapy and associated with increased risk of treatment failure [1, 2]. Although SNPs at positions 1034 and 1042 of *pfmdr1* were described as being related to phenotype modulation by altering a drug pocket in PfMDR1 [8], they are very infrequent in Africa.

ACT drug resistance has recently been reported at the Thai-Cambodian border [9–11]. Historical evidence shows that the emergence of drug-resistant *P. falciparum* strains first originated in Southeast Asia and then, spread to Africa [12]. East Africa has been a major focus of drug resistance spread, and development in sub-Saharan Africa [12, 13], probably originated at Southeast Asia [13–15].

Surveillance of changes in prevalence of *pfmdr1* SNPs may serve as an early warning tool of emerging *P. falciparum* tolerance/resistance to ACT [16]. Mozambique, an Eastern Africa country, with a population of 20.2 million, is amongst the ten most affected countries by malaria in the world. Here, malaria represents one of the major public heath challenges, being responsible for nearly 45% of registered disease episodes, 56% of cases admitted to paediatric health facilities and about 26% of deaths in hospitals [17]. During the last decade, African countries have changed first-line treatment of uncomplicated falciparum malaria to ACT due to the development of resistance to successively introduced anti-malarial drugs. In Mozambique, CQ was replaced by sulphadoxine-pyrimethamine (SP) in 2002 (in combination with AQ) and in 2004 AQ was replaced by artesunate (ATN) in that combination. Since 2009, this combination was replaced by artemether-lumefantrine (AL) [17, 18].

Apart from the work of Raman and colleges [19], the prevalence of *pfmdr1* different alleles in Maputo and Mozambique is still scarce, either after or before the introduction of ACT. In line with this, molecular markers related to *P. falciparum* susceptibility were analysed in samples from Maputo for the period before and after transition to ACT.

## Methods

### Biological samples

DNA samples included in this study were collected from patient blood spots obtained at three health facilities in Maputo area. One study was conducted at Hospital Central de Maputo, Centro de Saúde de Bagamoio and Centro de Saúde de Boane between June 2003 and June 2005 and the other at Centro de Saúde de Boane and Centro de Saúde 1° de Maio between March 2010 and March 2012. Both studies were reviewed and approved by the Ethical Committees of the Ministry of Health of Mozambique and informed consent was obtained at the time.

### Genetic characterization of the parasites

Included samples came from PCR-confirmed *P. falciparum* infections [20]. DNA was extracted from filter paper blood spots using Chelex as described elsewhere [21]. *Plasmodium falciparum* mutations in *pfmdr1* gene were typed by PCR-RFLP as described elsewhere [21, 22], primers sequences, amplification cycles and restrictions enzymes for 86Y and 1246Y are described in [21] and for Y184 in [23].

### Statistical analysis

Allele and haplotype prevalence in the groups were compared by Chi-squared tests and, when appropriate, a residual analysis was performed in order to determine reasons for the rejection of independency between the variables under study. Statistical significance was set at p ≤ 0.05. All calculations were performed with IBM SPSS 20.0.

## Results

In this study, the presence of alleles of the *pfmdr1* gene (codons 86, 184 and 1246) in patients with uncomplicated falciparum malaria, before (2003–2005) and after the implementation of ACT in Maputo area (2010–2012) were analysed. A total of 133 samples were included in the group 2003–2005 (only 56 of the 133 samples were analysed for the codon 184) and 351 in 2010–2012.

The typing efficiency for each codon was as follows: 2003–2005 group, 100% of samples for the N86Y, 100% for Y184F (only 56 of the 133 samples were analysed) and 91% for D1246Y; 2003–2005 group, 96.9% of samples for the N86Y, 99.1% for Y184F and 96.0% for D1246Y.

**Figure 1:**
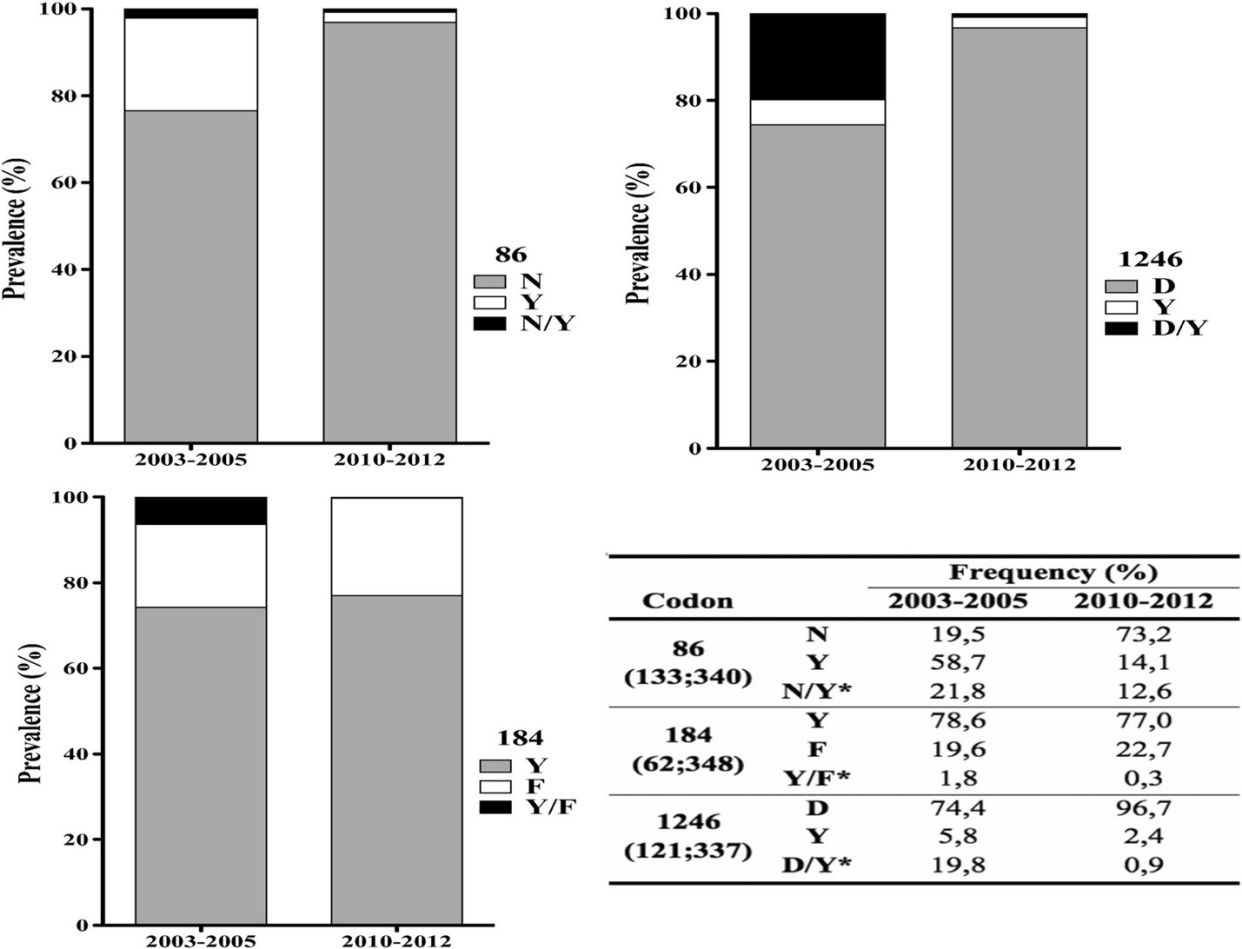
**Temporal changes of prevalence at codons 86, 184 and 1246.** Codons frequencies in each sample group are presented in the inserted table (numbers in brackets represent number of samples corresponding to 2003–2005, 2010–2012 and *representing mixed genotype infections).

The individual prevalence of SNPs at codons 86, 184 and 1246 in 2003–2005 and 2010–2012, including mixed codon infections, are shown in Figure 1 with Table 1 showing individual SNPs and haplotypes.

**Table 1:** **Prevalence of** ***Pfmdr1*** **haplotypes at codon N86Y, Y184F and D1246Y in Maputo, before and after the introduction of artemisinin-based combination therapy**

Codon	Haplotype	2003-2005*	2010-2012*
**86/184** (49;293)	**NF**	10.2	24.3
	**YF**	8.2	0.3
	**NY**	12.2	59.4
	**YY**	69.4	16.0
**86/1246** (77;286)	**ND**	27.3	84.4
	**NY**	1.3	0.3
	**YD**	64.9	13.6
	**YY**	6.5	1.7
**184/1246** (53;330)	**FY**	0.0	0.0
	**FD**	20.8	23.3
	**YY**	7.5	2.4
	**YD**	71.7	74.3
**86/184/1246** (45;282)	**NFD**	11.1	24.8
	**NFY**	0.0	0.0
	**NYY**	2.2	0.4
	**NYD**	11.1	59.2
	**YYY**	4.4	1.8
	**YFD**	8.9	0.4
	**YFY**	0.0	0.0
	**YYD**	62.2	13.5

*Sample collection period. Numbers in brackets represent number of samples collectedduring the correspondent periods 2003–2005;2010–2012.

To examine for temporal changes in prevalence of SNPs at codons 86, 184 and 1246, mixed infections (both alleles present) were analysed together with the polymorphism not associated with ACT tolerance/resistance, i.e., *pfmdr1* 86Y, Y184, 1246Y. The wild type allele N86 significantly recovered in prevalence from 19.5% in 2003–2005 to 73.2% 2010–2012 (p < 0.0001). Although allele 184F also increased in prevalence from 19.6% in 2003–2005 to 22.7% in 2010–2012, it was not statistically significant (p = 0.7300). The prevalence of allele D1246 also increased significantly from 74.4 to 96.7% in 2010–2012 (p < 0.0001). In 2010–2012, mixed infections were less frequent, a 58% decrease in multiple infections was observed for codon 86 and more than 80% for codons 184 and 1246 (Figure 1).

Haplotypes were compared on a two- and three-codon basis (Table 1) and analysed for temporal prevalence change. Minority haplotypes (<5%) and mixed infections were excluded from the analysis. After implementation of ACT (2010–2012), *pfmdr1* haplotypes, either two- and three-codon basis, were generally less diverse than before the implementation of ACT (2003–2005) (Table 1). From 2003–2005 to 2010–2012, haplotypes N86-184Y and N86-F184 significantly increased from 12.2 to 59.4% and 10.2 to 24.3%, respectively (p < 0.0001). On the contrary, 86Y-Y184 decreased from 69.4% in 2003–2005 to 16.0% in 2010–2012 (p < 0.0001) (Table 1). Comparison of the codons combination 86–1246 showed a significant increase of the N86-D1246 haplotype from 27.3% in 2003–2005 to 84.4% in 2010–2012 (p < 0.0001). Haplotype 86Y-D1246 of the same combination, decreased from 64.9 to 13.6% (p < 0.0001). Although the prevalence of haplotypes F184-D1246 and 184Y-D1246 increased from 2003–2005 to 2010–2012 (Table 1) it did not reach statistical significance (p = 0.7455). The three-codon basis haplotype analysis revealed that, except for NFD and NYD, all the other codon combination decreased in prevalence from 2003–2005 to 2010–2012 (Table 1). This tendency was highly significant for YYD (from 62.2 to 13.5%; p < 0.0001). Haplotypes YFY and NFY were not detected either in 2003–2005 or 2010–2012 samples. In 2010–2012, more than half of the infections carried the NYD, showing a significant trend of increase from 11.1 to 59.2% (p < 0.0001). The haplotype NFD (associated with the ability to withstand higher lumefantrine concentrations) also presented a significant increase in prevalence from 11.1% in 2003–2005 to 24.8% in 2010–2012 (p < 0.0001).

## Discussion

There are signs of decreasing malaria prevalence (5.168,684 cases in 2005 and 3.381,371 cases in 2010) in Mozambique, but the disease remains a major cause of morbidity and mortality [17, 18]. Continued surveillance of molecular markers of drug resistance and particularly, potential markers of ACT drug tolerance are important tools for the success of malaria control programmes.

In Mozambique, CQ was replaced by SP in combination with AQ in 2002, followed by SP + AS in 2004 and then SP + AS was replaced by AL as first line in 2009, because of the high levels of SP resistance. Nevertheless, from 2004 AL has been the second line of treatment of uncomplicated malaria in the country [17, 18]. In the present study, the prevalence and temporal changes of N86Y, Y184F and D1246Y *pfmdr1* alleles were studied in Maputo area.

The present study found a high prevalence of mutant type 86Y and wild-type Y184 in samples from 2003–2005 (58.7 and 74.3%, respectively). This is in accordance with other studies performed in Africa where 86Y and Y184 frequencies were also high before the introduction of ACT [3–5, 16, 24–30]. Decreasing prevalence of the 86Y after introduction of ACT in Mozambique was also observed during two recent studies from Inhambane and Gaza, Mozambique [19, 31]. In line with this tendency, a high prevalence of wild type N86 and D1246 and the mutant 184Y was detected in the 2010–2012 group of samples (Figure 1), which is again in accordance withother studies in Africa [3–5, 16, 24–31]. Overall, these results suggest that the prevalence of 86Y and Y184 was high when SP and AQ were in use on a large-scale basis, but when these drugs were substituted by ACT their prevalence decreased significantly, which might indicate that this haplotype does not confer a fitness advantage upon ACT pressure [32].

Before ACT implementation (2003–2005) the most common triple haplotype (*pfmdr1* 86,184 and 1246) was YYD (62.2%), indicating that the previous first-line treatment based in quinolines (i.e., CQ or AQ) mainly selected *pfmdr1* 86Y and 184Y. The second most common haplotypes were NYD and NFD (11.1%), indicating that N86 and D1246 were already being selected by the decreasing use of quinolines and the introduction of AS in combination with SP. This is in line with the observations that, AS *per se* potentially selects for *pfmdr1* N86 and D1246, which have been associated with decreased susceptibility to artemisinins *in vitro* [33, 34]. Importantly however, no such selection has been shown after monotherapy with artemisinin derivatives *in vivo*. Another reason for the increased N86 and D1246 prevalence might be that SNPs associated with AQ resistance (86Y and 1246Y) cause a fitness cost to the parasite [26], which would affect the selection pattern under different drug pressures. That is, when quinolines pressure was released, wild type alleles (N86, D1246) immediately begin to rise in the parasite population. The combination of SNPs 86Y-1246Y was rare in 2010–2012 group of samples (Table 1), suggesting again that they may be associated with a significant fitness cost as observed previously [26].

Five to seven years after implementation of ACT in Mozambique, a significant selection of NFD and NYD was observed in the Maputo area. Other studies with African-derived samples support the idea that haplotype N86, 184F and D1246 is AL selected [3–5], while 86Y, 184Y and 1246Y is AQ or CQ selected [2, 3, 35–37]. Additionally, a study from Thailand suggested that selection of the haplotype N86-184F was likely caused by AS [38]. Generally, these results are consistent with a recent study performed in samples from people living in a touristic area in Inhambane, Mozambique [31] with those from numerous studies in Africa [3–5, 16, 29, 30, 39].

Despite the challenges of validating candidate markers of drug resistance, namely for ACT (a combination of chemically unrelated molecules) [10, 40–43], several works point to *pfmdr1* SNPs as possible modulators for ACT response mainly based on the partner drug [2–5, 8, 23, 28–31, 35, 42]. In the absence of definite marker for artemisinin (ART) resistance, molecular monitoring of its partner drug markers may contribute to predict the effectiveness of ACT. Currently, the K13-propeller mutations (recently proposed by Ariey and colleagues [44] as a molecular marker for ACT resistance) may be a useful marker for large-scale surveillance efforts to contain and prevent global spread of ACT resistance.

## Conclusion

A sustained success in malaria control is strongly dependent on continued effectiveness of first line treatment. The results of the present work are in line with data found recently in other African countries, where the wild type allele N86 showed increased prevalence after introduction of AL. Furthermore, it is shown a temporal increase of 184F mutant type after introduction of AL, and a significant increase of the triple haplotype N86-184F-D1246. Therefore, there is cause for concern/attention and close continued surveillance of *pfmdr1* SNPs*,* which remain highly relevant as a marker of reduced susceptibility to AL.
